# Material characterization of GPX^®^: A versatile *in situ* solidifying embolic platform technology

**DOI:** 10.3389/fbioe.2023.1095148

**Published:** 2023-01-16

**Authors:** Russell J. Stewart, Monika Sima, Jessica Karz, Joshua P. Jones

**Affiliations:** ^1^ Department of Biomedical Engineering, University of Utah, Salt Lake City, UT, United States; ^2^ Fluidx Medical Technology, Inc., Salt Lake City, UT, United States

**Keywords:** embolization, embolic agent, liquid embolic, chemoembolization (TACE), doxorubicin, polyelectrolytes

## Abstract

Endovascular embolization is a minimally invasive procedure during which blood flow to targeted tissues is selectively occluded. The list of clinical indications for embolization continues to expand. Liquid embolic agents are injectable compositions that transition into a solid or semi-solid form when introduced into blood vessels. The mechanism that triggers the liquid-to-solid transition is a key distinguishing feature of liquid embolic agents. GPX is a waterborne liquid embolic agent comprising oppositely charged polyelectrolytes: polyguanidinum and inorganic polyphoshate. *In situ* solidification is driven by electrostatic condensation of the polyelectrolytes, triggered by ionic strength differentials. We report *in vitro* characterization of the material properties of GPX, it is *in vivo* effectiveness in acute animal studies, and its potential for chemoembolization. The viscosity of GPX can be varied over a wide range by adjusting the polyguanidinium MW and/or concentration. Formulation of GPX with either tantalum microparticles (30 wt%) or iodinated radiocontrast agents (300 mgI ml^−1^) did not significantly change the flow behavior of GPX; the viscosity was independent of shear rate and remained within a clinically practical range (80–160 cP). Formulation of GPX with doxorubicin substantially increased viscosity at low shear rates and resulted in a power law dependence on shear rate. High contrast and effective vascular occlusion were demonstrated in both swine kidneys and rete mirabile. Contrast from iodinated compounds was temporary, dissipating within hours. The doxorubicin *in vitro* release profile was linear over 90 days. The results demonstrate that GPX is a versatile liquid embolic platform that can be formulated with a wide range of viscosities injectable at clinically practical flow rates, with either transient or permanent contrast, and that can provide prolonged zero-order delivery of doxorubicin to embolized tissues.

## 1 Introduction

Endovascular embolization is a minimally invasive procedure during which transcatheter delivery of embolic devices or agents, guided by real-time imaging, block blood flow to targeted tissues with therapeutic intent (Hu et al., 2019). Medical conditions treatable by endovascular embolization are diverse and continue to expand (Dariushnia et al., 2021). Broad categories include occlusion of vascular malformations or other vascular and lymphatic abnormalities (Wu et al., 2019; Vollherbst et al., 2022), control of acute bleeding due to disease, trauma, or surgery (Loffroy and Guiu, 2009; Jeske et al., 2010; Lopera, 2010), and devascularization of benign and malignant tumors (Ginat et al., 2009; Lazzaro et al., 2011; Bester et al., 2014; Talenfeld et al., 2014). In the case of malignant tumors, embolization has been combined with chemotherapeutic agents, a procedure referred to as transarterial chemoembolization (TACE), to simultaneously occlude blood flow and provide locoregional delivery of drugs to targeted tumor tissue, avoiding complications of systemic drug delivery (Inchingolo et al., 2019). Embolics can be classified into three broad groups: 1) solid devices, such as metallic or polymeric coils, particles, and microspheres that form mechanical blockades; 2) soft gelatin gels that deform during delivery and return to a soft gel state at the target site [Embocube^®^, GEM™ (Albadawi et al., 2020)]; and, 3) liquid agents that are low viscosity solutions that transition into a semi-solid form when delivered into a blood vessel that occludes blood flow. Liquid embolic agents are the focus of this report.

Liquid embolic agents are distinguished, in large part, by the *in situ* solidification or gelation mechanism. The liquid-to-solid transition with a clinically practical trigger mechanism is one of the biggest technological challenges in the development of liquid embolic agents. Liquid embolics in current clinical use are categorized as polymerizing or precipitating. Polymerizing embolics are liquid monomers of alkyl esters of cyanoacrylates, such as N-butyl-2-cyanoacrylate (NCBA, e.g., Trufill™). Rapid polymerization into a solid mass is initiated when the cyanoacrylate monomers contact hydroxyl ions in blood. Precipitating embolics are solutions of water-insoluble, ethylene-vinyl alcohol copolymers (pEVOH) dissolved in the water-miscible organic solvent, dimethyl sulfoxide (DMSO). Variations include Onyx™, PHIL™, and Squid™ (Vollherbst et al., 2022). When introduced into an aqueous environment (blood), DMSO diffuses out of the solution resulting in outside-in precipitation of pEVOH into a semi-solid occlusion (Taki et al., 1990; Pollak and White, 2001; Lubarsky et al., 2010).

The diverse indications for endovascular embolization and limitations of current liquid embolic agents, primarily related to toxicity and handling characteristics, drive continuing development of new embolic materials. Liquid embolics in various stages of pre-clinical development comprise a wide range of natural and synthetic polymeric components and exploit a variety of *in situ* solidification mechanisms. Two-component systems rely on mixing separate precursors prior to delivery or at the delivery site to initiate an *in situ* liquid-to-solid transition. Two-component systems therefore require packaging in two separate syringes and in most cases delivery through dual-lumen or coaxial microcatheters. Examples of two-component systems include sodium alginate (Becker et al., 2005) and inorganic polyphosphate (Momeni et al., 2016) solutions that gel or precipitate when mixed with a solution of divalent Ca^2+^ ions co-delivered to the *in situ* target site. Other two-component systems comprise reactive precursors that covalently crosslink into solids when mixed. One such system, referred to as PPODA-QT, comprises a solution of two hydrophobic monomers, poly (propylene glycol) diacrylate (PPODA) and pentaerythritol tetrakis (3-mercaptopropionate) (QT), which are crosslinked into a solid through Michael addition of the thiol groups to the acryloyl groups. Crosslinking is initiated when a high pH aqueous solution is emulsified into the solution of hydrophobic monomers immediately prior to injection through a microcatheter (Riley et al., 2011; Huckleberry et al., 2021). Another two-component system, Embrace™, comprises a solution of polyethylene glycol (PEG) diacrylate monomers and a solution of an organic peroxide free radical initiator. The separate solutions are delivered through a coaxial microcatheter. Mixing of the two reactive components at the target site results in crosslinking of PEG into a hydrogel by free radical polymerization through the acryloyl groups (Kauvar et al., 2021a; Gandras et al., 2022). A category of single component formulations (not counting potential contrast agents) rely on a temperature induced solidification mechanism. This category of liquid embolics comprise amphiphilic copolymers that are designed to be liquid solutions at low temperature (ca. 20°C) and solidify through hydrophobic interactions at elevated body temperatures (ca. 37°C). Early temperature-dependent formulations were based on N-isopropyl-acrylamide-N-propylacrylamide-vinyl pyrrolidone copolymers (Li et al., 2005). Additional examples include formulations based on chitosan/β-glycerophosphate (Wang et al., 2011) and silk elastin-like proteins (Poursaid et al., 2015).

**FIGURE 1:**
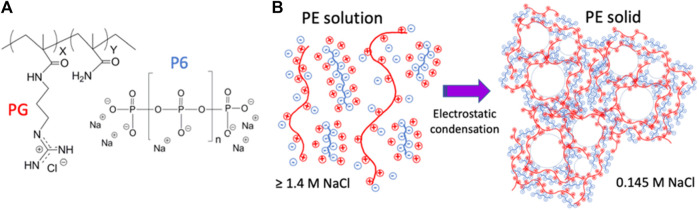
**(A)**) Polyelectrolyte components of GPX. PG: chloride salt of N-(3-guanidinopropyl) methacryamide-co-methacrylamide. P6: sodium salt of hexametaphosphate. **(B)**) GPX liquid-to-solid transition mechanism. In solution, counter-ion shielding prevents macro-ion association. In low ionic strength environments, counter-ion diffusion leads to macroion association and solidification.

GPX is a waterborne liquid embolic agent that solidifies *in situ* through electrostatic condensation of oppositely charged polyelectrolytes (Figure 1B). GPX is an aqueous solution of the chloride salt of a synthetic polyguanidium (PG) and the sodium salt of an inorganic polyphosphate (Figure 1A). Early GPX prototypes have been previously reported (Jones et al., 2016; Fries et al., 2022). The guanidinyl sidechains of the polyguanidiums are strong bases (pK_a_ ∼13) (Fitch et al., 2015) and the phosphate subunits of the polyphosphate are strong acids (pK < 4). The polyelectrolytes are therefore fully charged at any physiologically relevant pH. The high monovalent counterion concentrations in the solutions (>1.4 M Na^+^ and Cl^−^) are sufficient to prevent the oppositely charged polyelectrolytes from associating, resulting in stable, low viscosity, single-phase solutions. When liquid GPX is introduced into a lower ionic strength environment, blood for example, the monovalent counterions diffuse out of the solution, the oppositely-charged polyelectrolytes associate electrostatically, and the composition rapidly transitions into a microporous solid from the outside in (Figure 1B). Here, we report *in vitro* characterization of the material properties of liquid and solid GPX, as well as GPX formulated with contrast agents and doxorubicin (a chemotherapeutic agent), *in vivo* embolic effectiveness in acute large animal pilot studies, and *in vitro* kinetics of doxorubicin release.

## 2 Results and discussion

### 2.1 Liquid GPX viscosity and injection pressures

**FIGURE 2:**
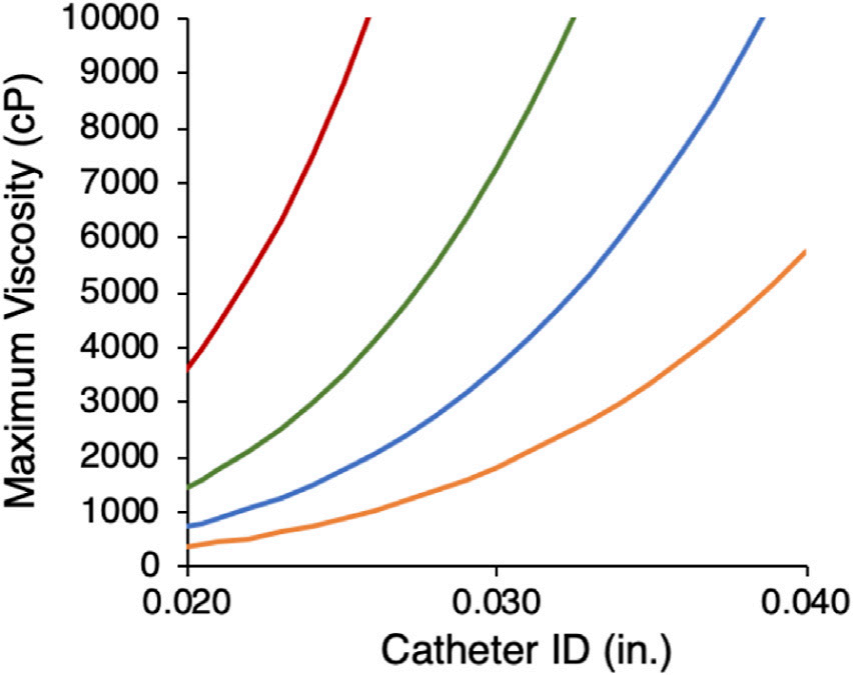
Calculated maximum viscosity limits for microcatheters with 800 psi burst ratings as a function of microcather ID at four flow rates (mL min^−1^): 1.0 (orange), .5 (blue), .25 (green), .1 (red).

In practice, liquid embolics are delivered through microcatheters that can be as long as 175 cm and with internal diameters (ID) as small as .015 inches (.38 mm) (Evtoday, 2022). A critical design criterion of liquid embolics, therefore, is the viscosity of the delivered form, which determines the dimensions of the microcatheters through which a liquid embolic can be delivered at clinically useful flow rates and at injection pressures that are within the physical limits of the microcatheter (burst pressure) and the operator (manual strength). The injection pressure required for steady flow of a liquid embolic agent is determined by four factors; flow rate, viscosity, microcatheter length and ID. These factors are related by the Hagen-Poiseulle equation for steady laminar flow of a Newtonian fluid through a cylindrical tube:

$$∆P=\frac{8Q\mu L}{{\pi r}^{4}}$$

where *P* is pressure, Q is the volumetric flow rate, *μ* is viscosity, *r* is the radius, and *L* is the length of the microcatheter. Burst pressures for commercial microcatheters range from 300 to 1,200 psi. Using a burst rating of 800 psi and length of 150 cm as a clinically relevant illustrative example, the calculated maximum liquid embolic viscosity to avoid microcatheter failure was plotted for a range of flow rates and IDs (Figure 2). For large catheters (≥.040″ ID) at an injection rate of 1.0 mL min^−1^, the injection pressure for viscosities as high as 5000 cP (Pa·s = cP/1,000) remain below the microcatheter burst pressure. As microcatheter diameter is decreased, maximum viscosity decreases rapidly, reflecting the inverse fourth power relationship of pressure to microcatheter radius. For neurovascular microcatheters with ID ≤ .015″, the viscosity would have to be <70 cP for delivery at 1 mL min^−1^. These calculations apply only to Newtonian fluids whose viscosity does not depend on shear rate. The wide range in microcatheter dimensions, dictated by the embolization site, and practical lower limits on injection rate, suggest that the ability to adjust viscosity over a wide range is a desirable property of a liquid embolic technology platform. The effect of several formulation parameters on the viscosity of liquid GPX were evaluated and described in the following sections.

#### 2.1.1 Polyguanidinium molecular weight and concentration

**FIGURE 3:**
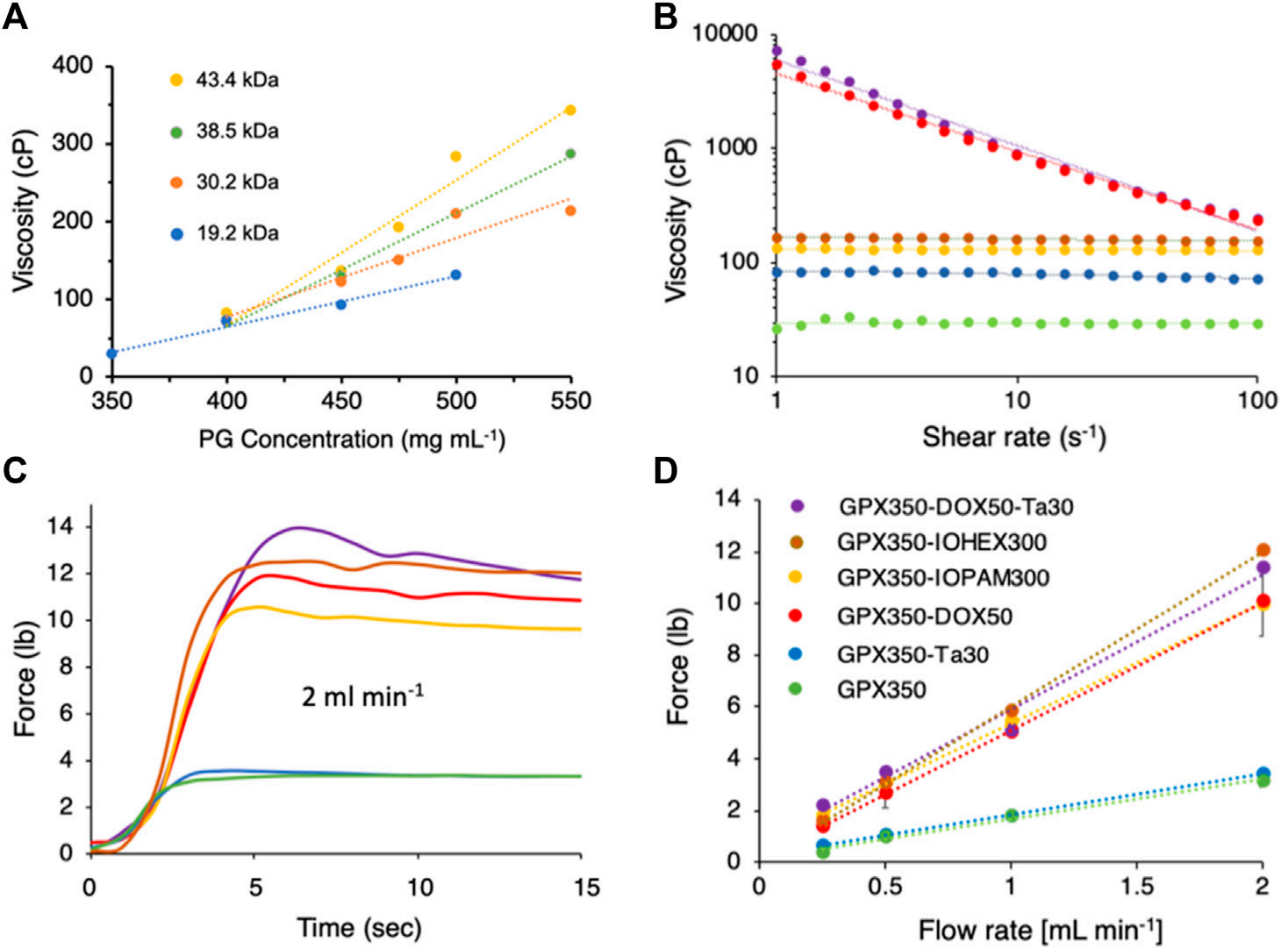
**(A)** GPX viscosity as a function of polyguanidium (PG) MW and concentration. Dotted lines are linear fits to the data points. **(B)** Flow curves of five GPX formulations. The colors and symbols corresponding to the formulations are shown in panel D. Data points represent the mean of three measurements. Dotted lines are linear fits to the data points. Error bars were omitted for clarity. **(C)**. Representative time courses of injection forces required for each formulation injected through a 120 cm, .025″ ID microcatheter at 2 mL min^−1^. **(D)**. Injection force as a function of flow rate. Data points represent the mean of three measurements. Error bars represent ± 1 SD.

GPX was prepared with PG-HCl_n_ copolymers (Figure 1A) with a range of MWs (19.2–43.5 kDa) and concentrations (350–550 mg mL^−1^). All GPX solutions were prepared with Na_n_MP (Figure 1A) at a 1:1 polymeric charge ratio. As expected, the viscosity increased with both PG MW and concentration (Figure 3A). The 10-fold increase in GPX viscosity over this narrow range demonstrated that PG-HCl_n_ concentration and/or MW can be adjusted to tune GPX viscosity for delivery through a wide array of microcatheters. The range of viscosities can be further extended by using a wider range of PG MW and polyelectrolyte concentrations. GPX350 (where the number refers to the PG concentration) was used as the base formulation to evaluate the effect of additional components on GPX viscosity and injectability.

#### 2.1.2 Contrast agents

Embolization procedures are generally done using fluoroscopic imaging to guide precise placement of the embolic agent. Therefore, another important design criterion for liquid embolic agents is the incorporation of radiocontrast agents. Contrast agents can be permanent or temporary with advantages and disadvantages for both. Permanent radiopacity provides high contrast imaging of the delivery site both during and after the procedure. However, these agents remain in the subject indefinitely and can cause imaging artifacts in subsequent CT scans. There is also potential for undesirable discoloration, tattooing, if the contrast agent is located under skin. And, permanent metallic contrast agents, such as tantalum, can cause sparking if electrocautery is used in subsequent surgeries near the embolization site. Conversely, contrast agents that are immediately carried away from the embolization site, such as when embolic beads or particles are suspended in a contrast medium carrier, do not allow previously delivered beads or particles to be visualized if additional injections are necessary. A mechanism to provide temporary contrast that persists for at least the length of the embolization procedure, but that disappears within hours or days afterwards would avoid the disadvantages of both short-lived and permanent contrast agents. We demonstrated that GPX can be effectively formulated with either permanent or temporary radiocontrast.

##### 2.1.2.1 Metallic contrast agents

For permanent contrast, 30 wt% Ta microparticles were mixed into GPX350 (GPX350-Ta30). Tantalum microparticles at concentrations up to 30 wt% are routinely included in commercial liquid embolic agents such as Onyx™ and Trufill™ as contrast agents. Flow curves (viscosity vs. shear rate) for both formulations are shown in Figure 3B. GPX350 and GPX350-Ta30 both behaved as Newtonian fluids; their viscosity was independent of shear rate over the range 1–100 s^−1^. The addition of 30 wt% Ta microparticles increased the viscosity of GPX350 from about 30 to 80 cP (Figure 3B). The viscosity of GPX350-Ta30 remained within a range suitable for the smallest ID microcatheters at clinically useful flow rates. The force required to inject the formulations through a 120 cm, .0235 “(.6 mm) ID stainless steel microcatheter was determined by loading the formulations into 1 ml syringes [bore diameter .195” (.5 cm)] and injecting the solutions at fixed flow rates using a syringe pump fitted with a compression load cell. The addition of 30 wt% Ta microparticles did not significantly increase the force required to push GPX350-Ta30 through the microcatheter, 3.4 lbs versus 3.2 lbs for GPX350 at 2.0 mL min^−1^ (Figures 3C, D).

##### 2.1.2.2 Iodinated contrast agents

For temporary contrast, GPX350 was mixed with the iodine containing compounds, iohexol (GPX350-IOH300) and iopamidol (GPX350-IOP300) at 300 mgI ml^−1^. Iohexol and iopamidol are the iodine-based contrast agents in commercial Visipaque™ and Isovue™, respectively. Both formulations behaved as Newtonian fluids over the shear rate range 1–100 s^−1^ (Figure 3B). The viscosities of GPX350-IOP300 and GPX350-IOH300 were 130 and 160 cP, respectively, versus 80 cP for GPX350-Ta30, with a concomitant increase in injection forces (Figures 3C, D).

##### 2.1.2.3 Doxorubicin

Doxorubicin HCl (DOX) is an FDA approved anthracycline antibiotic with antineoplastic activity that is used to treat a broad range of malignancies. DOX has been emulsified with iodized oil (Lipidol) for transarterial chemoembolization (TACE) since the 1980s (Ryusaku Yamada et al., 1983; Nakamura et al., 1989). DOX and other agents have also been loaded into calibrated microspheres for TACE (Perez-Lopez et al., 2022). To evaluate its potential for TACE, GPX350 was prepared with 50 mg mL^−1^ of DOX (GPX350-DOX50) and 50 mg mL^−1^ DOX with 30 wt% Ta microparticles (GPX350-DOX50-Ta30). The DOX formulations shear-thinned; viscosity had a power law dependence over the shear rate range 1–100 s^−1^ (Figure 3B). The flow behavior indexes (n) for GPX350-DOX50-Ta30 and GPX350-DOX50 were .92 and .93, respectively. An index value of n < 1 indicates shear thinning behavior.

The shear thinning behavior of the DOX formulations are also reflected in their injection force profiles (Figures 3C, D). Despite the substantially higher viscosities of the DOX formulations at low shear rates, the injection forces were similar to the iohexol and iopamidol formulations. The wall shear rate in an .025″ ID microcatheter is 100 s^−1^ at a volumetric flow rate of .12 mL min^−1^. Even at this low flow rate, the DOX formulations have thinned to viscosities approaching those of the iohexol and iopamidol formulations, a 20 to 30-fold reduction from the 1 s^−1^ viscosity (Figure 3B). The rapid drop in viscosity as the DOX formulations begin to flow accounts for the low injection forces. Importantly, the maximum injection force for all five formulations at all measured flow rates were well within the limits of any operator. The average palmer grip force has been reported as 23.4 lbs for men and 16.3 lbs for women (Mathiowetz et al., 1985).

The substantially higher viscosities at low shear rates (1 s^−1^) of the GPX350-DOX50 (5370 cP) and GPX-DOX50-Ta30 (7200 cP) formulations compared to the base formulation GPX350 (27 cP), demonstrated that DOX interacts with the polyelectrolyte components to increase cohesiveness of the solutions. Doxorubicin is an aromatic molecule (543.5 g mol^−1^) with a single positive charge at neutral pH. Likewise, the cationic guanidinium sidechains of PG are planar with six delocalized π electrons and have aromatic character referred to as Y-aromaticity (Gund, 1972). DOX can interact with the guanidinium sidechains of PG through cation-π, or π-π interactions. Indeed, DOX and a polyguanidinium have been shown to self-assemble into nanocomplexes through π interactions (Cui et al., 2018; Zhuang et al., 2019) and DOX can be loaded onto carbon nanotubes through π-π stacking (Liu et al., 2009). The shear thinning behavior of the DOX-containing GPX formulations may be due primarily to reversible, shear-induced disruption of DOX-guanidinium π interactions.

### 2.2 Solid GPX material properties

**FIGURE 4:**
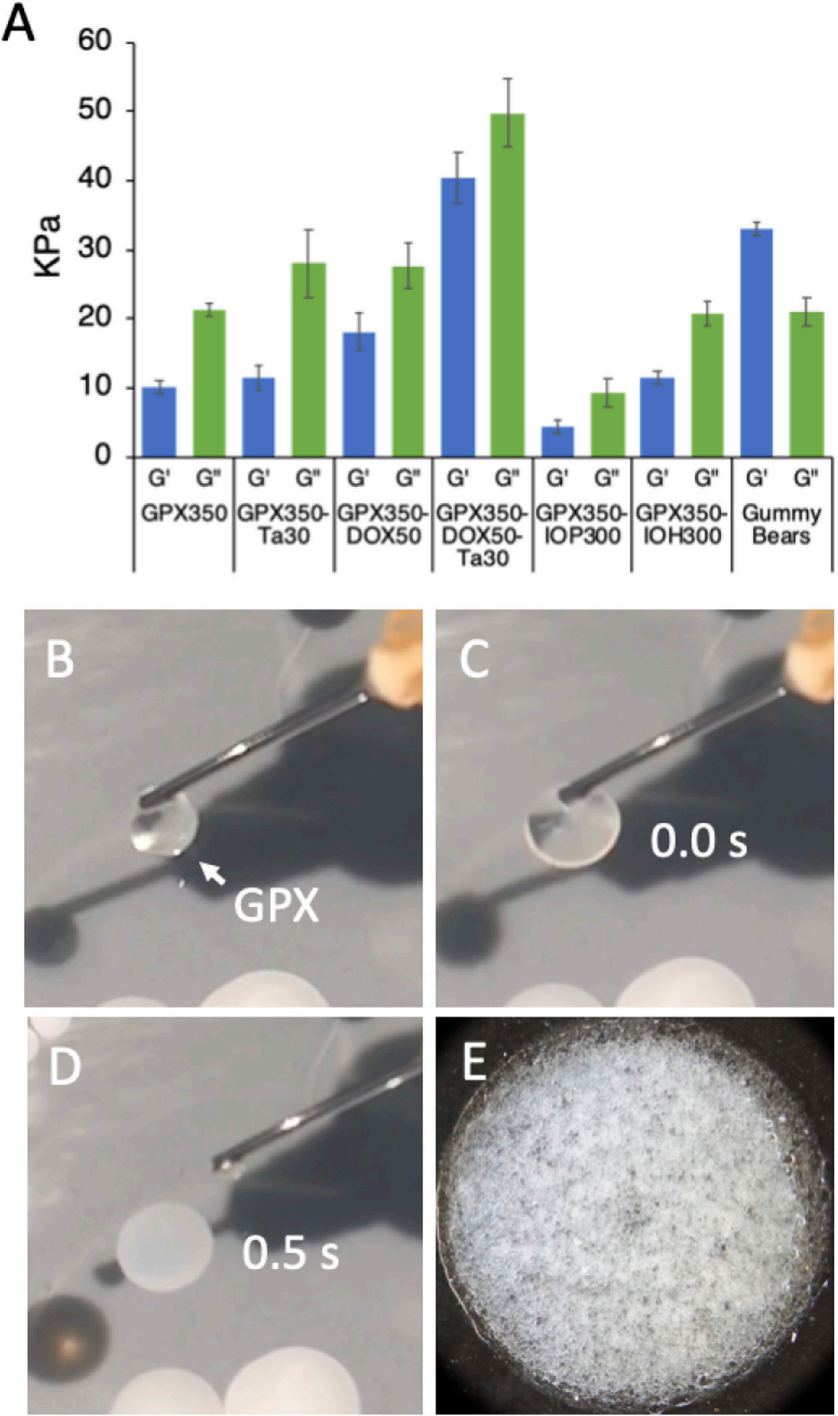
**(A)** Storage (G′) and loss (G″) moduli of the solid form of all five GPX formulations. Gummy bears were included as a familiar reference point. *n* = 3, error bars represent ± 1 SD. **(B)** Clear liquid form of GPX suspended on the tip of a cannula. **(C)** Initial contact of liquid GPX with surface of .9% saline solution. **(D)** .5 s (15 frames) after contact. Real time GPX solidification is shown in SVid 1. **(E)** Microporous solid form of GPX.

When the high ionic strength liquid form of GPX contacts a solution with physiological ionic strength it rapidly transitions into a microporous solid form (Figure 1B). This is demonstrated in the photos in Figure 4 B-E and in (Supplementary Video S1). GPX is a clear homogeneous solution (Figure 4B) that begins to transition immediately on contact with physiological saline (Figure 4C) and within .5 s (15 frames) has become a white solid membrane on the surface of the saline solution (Figure 4D). The microporous structure is apparent at higher magnification (Figure 4E). The elastic (G′) and viscous (G″) moduli of the solid were determined by oscillatory rheometry (Figure 4A). All five formulations are stiff viscoelastic solids with viscous (loss) moduli greater than the elastic (storage) moduli. To provide a familiar reference point for stiffness of the material, Haribo^®^ gummy bears were run under the same conditions. Solidified GPX350-DOX50-Ta30 is stiffer than a gummy bear. The consistency of GPX-Ta30 in an explanted swine renal artery 30 min after deployment is shown in Supplementary Video S2. The results demonstrate the material properties of solidified GPX are more than adequate to produce effective and stable occlusions of blood vessels. For comparison, natural fibrin clots have moduli of around 600 Pa. The stiffness of solidified GPX is at least an order of magnitude higher than several other embolic systems (Li et al., 2005; Avery et al., 2016; Poursaid et al., 2016; Zhou et al., 2016).

### 2.3 Embolic efficacy *in vivo*

#### 2.3.1 Swine kidneys

**FIGURE 5:**
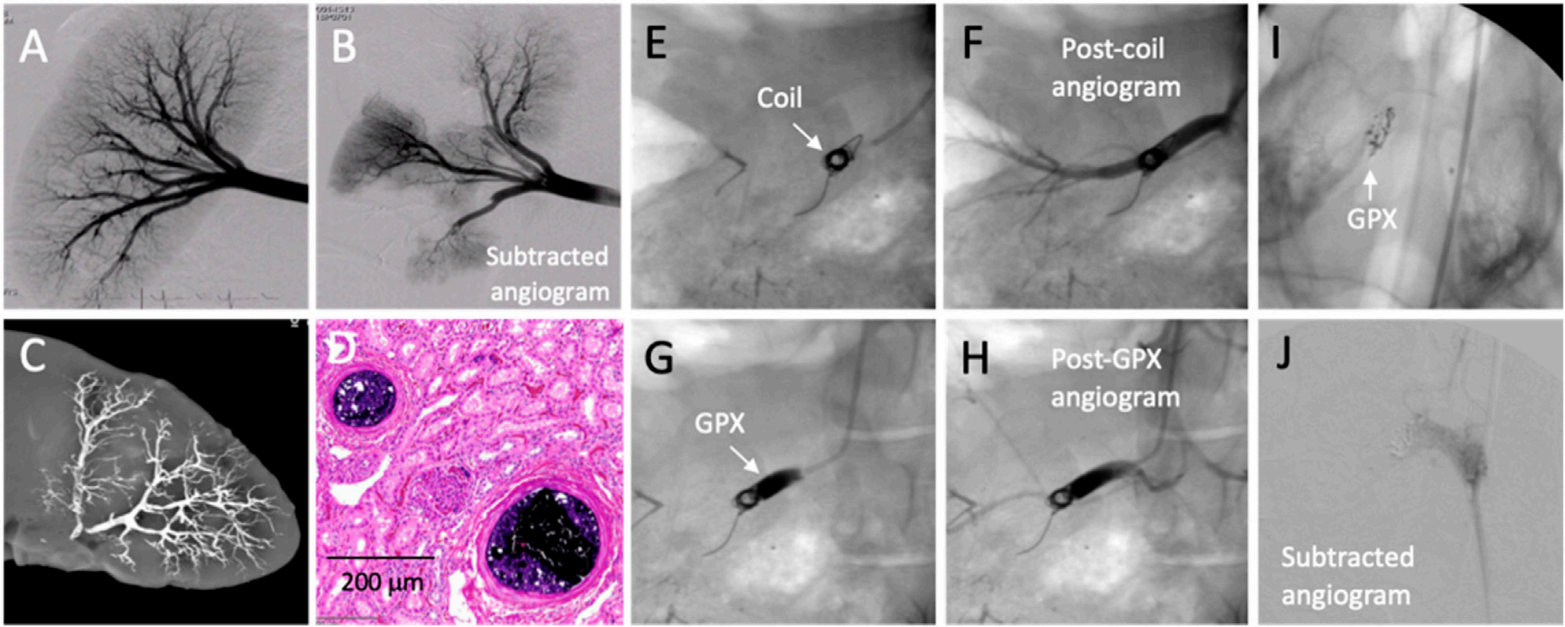
*In vivo* testing of GPX in acute swine models. **(A)** Pre-embolization angiogram. **(B)** Post-embolization subtracted angiogram after GPX400-Ta30 was sequentially injected into two separate renal artery sub-branches. **(C)** X-ray image of explanted embolized kidney. **(D)** Acute H&E stained histological section showing occluded arterioles. **(E)** Platinum coil in renal artery. **(F)** Post-coil angiogram. **(G)** GPX500-Ta30 deployed proximal to coil. **(H)** Post-GPX angiogram showed complete occlusion. **(I)** GPX350-Ta30 injected into right swine rete mirabile. **(J)** Post-embolization angiogram.

Preliminary non-GLP pilot studies were conducted in domestic swine, a common model for testing of new embolic devices and agents. A preembolization angiogram of a swine kidney is shown in Figure 5A. After accessing a renal artery through the femoral artery, GPX400-Ta30 was selectively delivered to a sub-branch artery in the caudal pole through a 2.8 F (.027″ ID) microcatheter under fluoroscopic imaging. The same microcatheter was then repositioned for deployment of GPX400-Ta30 into a second renal artery sub-branch. Subtraction angiography following the second injection demonstrated selective and complete occlusion of both renal artery sub-branches (Figure 5B). There was no adhesion to the microcatheter or clogging during the multiple deployments. During one procedure the tip of the microcatheter was deliberately left embedded in the deployed GPX for 2 min before withdrawal with no evidence of adhesion to the microcatheter tip. X-ray imaging of a resected kidney revealed excellent distal penetration and complete devascularization (Figure 5C). Sectioning of the embolized kidney tissue showed complete occlusion extending throughout the arterial tree from the hilus to small arterioles of the renal cortex (Figure 5D). The results of chronic (180 days) GLP embolization studies in swine and other large mammals will be described elsewhere.

#### 2.3.2 GPX combined with metallic coils

For proximal occlusion of larger blood vessels, higher viscosity GPX-Ta30 can be used in conjunction with embolic coils. The placement of a single 8 mm × 20 cm platinum coil in a swine renal artery (Figure 5E) failed to fully impede blood flow, as evident from post-coil angiography (Figure 5F). Application of GPX500-Ta30 proximal to the coil through a 4F (.041″ ID) microcatheter (Figure 5G) successfully occluded blood flow through the coil as evident from subsequent angiography (Figure 5H). There was no evidence of coil migration or GPX fragmentation following deployment. Advantages of adjunctive deployment of GPX with coils may be a significant reduction in the number of coils required to achieve full occlusion with a consequent reduction in the procedure time. Second, coils rely on intrinsic blood clotting and thrombosis. When used alone, coils are associated with less successful clinical outcomes in patients with coagulopathies (Loffroy et al., 2009; Kauvar et al., 2021b). Since vascular occlusion with GPX does not depend on thrombus formation co-deployment of GPX may improve outcomes of coil embolization in patients with coagulation disorders.

#### 2.3.3 Swine rete mirabile

Arteriovenous malformations (AVMs) are congenital defects that consist of a nidus of vessels that shunt blood from arteries to veins without an intervening capillary bed. AVMs are commonly embolized, often with liquid embolic agents, to mitigate the risk of rupture and a hemorrhagic stroke in the case of neural AVMs. The swine retia mirabile (RM) are a pair of microvascular networks situated at the termini of both ascending pharyngeal arteries prior to the circle of Willis. Due to their similar size and structure, swine RM has been used as a model for embolization of human neural AVMs (Massoud et al., 1994; Murayama et al., 1998; Wakhloo et al., 2005). The right rete of a swine was accessed through the femoral artery and GPX350-Ta30 was delivered into the RM using a .017″ ID microcatheter (Medtronic Echelon 10) positioned approximately 1 cm proximal to the RM. The GPX350-Ta30 completely filled the rete back to the tip of the microcatheter, but did not penetrate beyond the rete into the circle of Willis (Figure 5I). Complete occlusion of the right rete was confirmed by angiography (Figure 5J). The swine RM pilot study demonstrated that the low-viscosity, high-contrast version of GPX may be suitable for embolizing AVMs.

### 2.4 Temporary contrast

#### 2.4.1 Duration of non-ionic contrast agents in solid GPX

**FIGURE 6:**
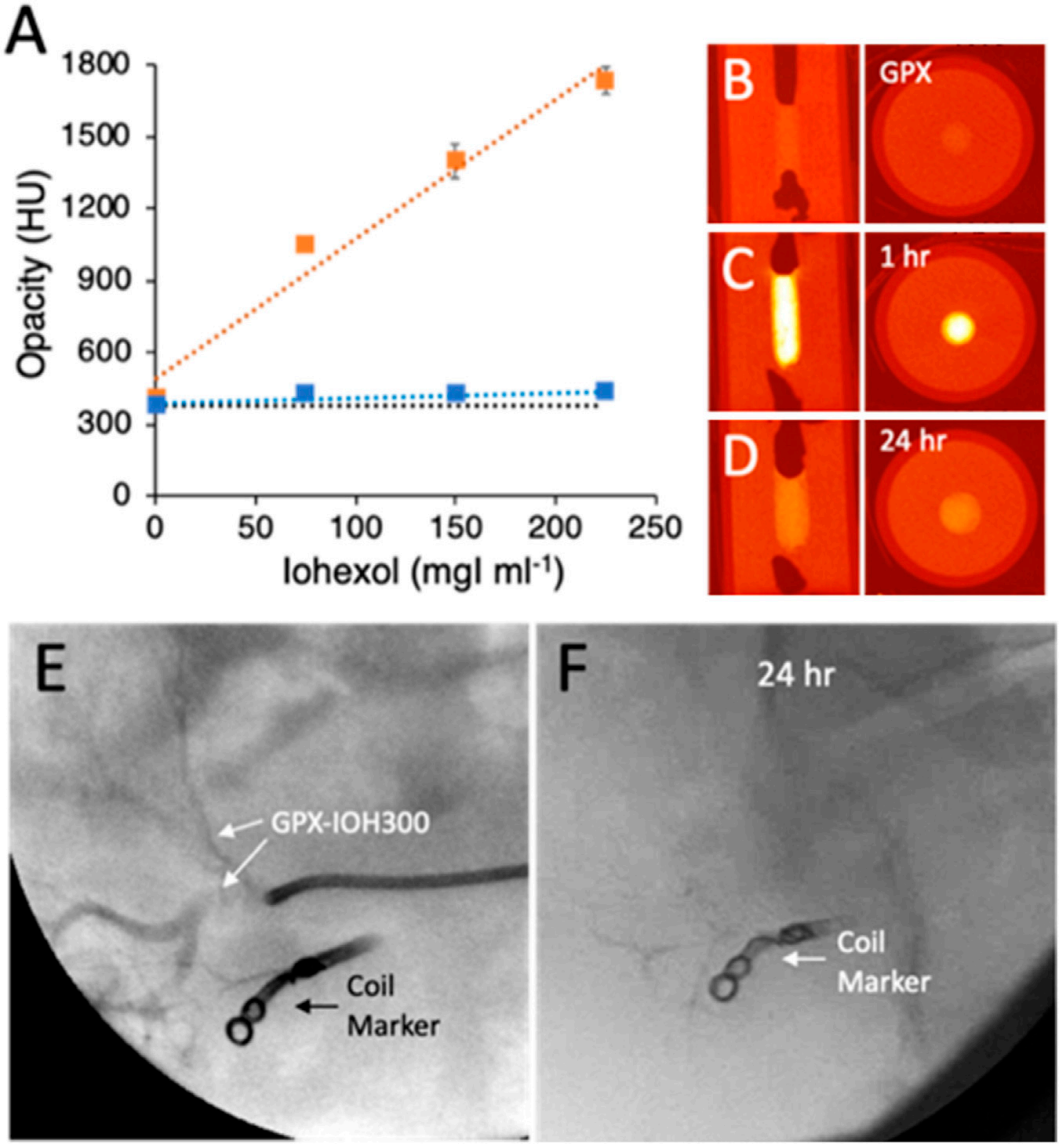
**(A)** Radiopacity of GPX350 with increasing concentrations of iohexol in gelatin tissue phantoms, after 1 h (orange symbols) and 24 h (blue symbols). Data points are the mean of three measurements and error bars represent ± 1 SD. Dotted lines are linear fits to the data points (*R* ^2^ ≥ .95). The black dotted line indicates the background opacity of GPX350. **(B)** Radial and axial microCT images of tissue phantoms containing GPX350. **(C)** GPX350-IOH75 after 1 h. **(D)** After 24 h. **(E)** Injection of GPX350-IOH300 into swine renal artery. **(F)** GPX350 was no longer visible in a fluorogram 24 h post-embolization.

The time course of non-ionic contrast agents diffusing out of solid GPX were evaluated *in vitro* by micro-CT in cylindrical gelatin tissue phantoms (Figure 6). Contrast-containing GPX400 solutions were prepared by dissolving dry PG-HCl_n_ (400 mg mL^−1^) and Na_n_MP at 1:1 polymeric charge ratios in iohexol solutions diluted with DI water to concentrations of 0, 75, 150, and 225 mgI mL^−1^. The GPX-IOH solutions were solidified in a cylindrical pore in the phantoms and imaged by micro-CT within 1 h of preparation and again after 24 h. Representative vertical and axial images of the phantoms containing 75 mg mL^−1^ iohexol are shown in Figures 6B–D. Radiopacity in Hounsfield Units (HU) of each sample at both time points are plotted in Figure 6A. Initial radiopacity increased linearly with iohexol concentration from background levels of 376 HU in gelatin phantoms containing GPX with no contrast to 1734 HU in phantoms containing 225 MgI ml^−1^ iohexol. As a reference point, a mid-range value for cortical bone is around 1100 HU. When re-imaged after 24 h, the radiopacity (423–433 HU) had decreased to near the background level of samples without iohexol (376 HU). Similar results were obtained with iodixanol (not shown). The results demonstrated that non-ionic iodinated contrast agents were highly visible after 1 h, but had largely diffused out of solid GPX into the surrounding tissue phantom within 24 h.

The temporary contrast time course was also evaluated *in vivo*. A metallic coil was placed in a subbranch of a swine renal artery to serve as a fiduciary maker. GPX400-IOH300 (.3 mL) injected into a second nearby subbranch of the renal artery under fluoroscopy was readily visible penetrating into the vasculature (Figure 6E). Complete occlusion of the artery was confirmed by angiography. Follow-up fluoroscopic imaging 24 h post-embolization revealed that the contrast agent had diffused out of the GPX and was no longer visible in the occluded artery (Figure 6F). Angiography 7 days post-embolization confirmed that the target region remained fully occluded.

#### 2.4.2 Doxorubicin release kinetics *in vitro*

**FIGURE 7:**
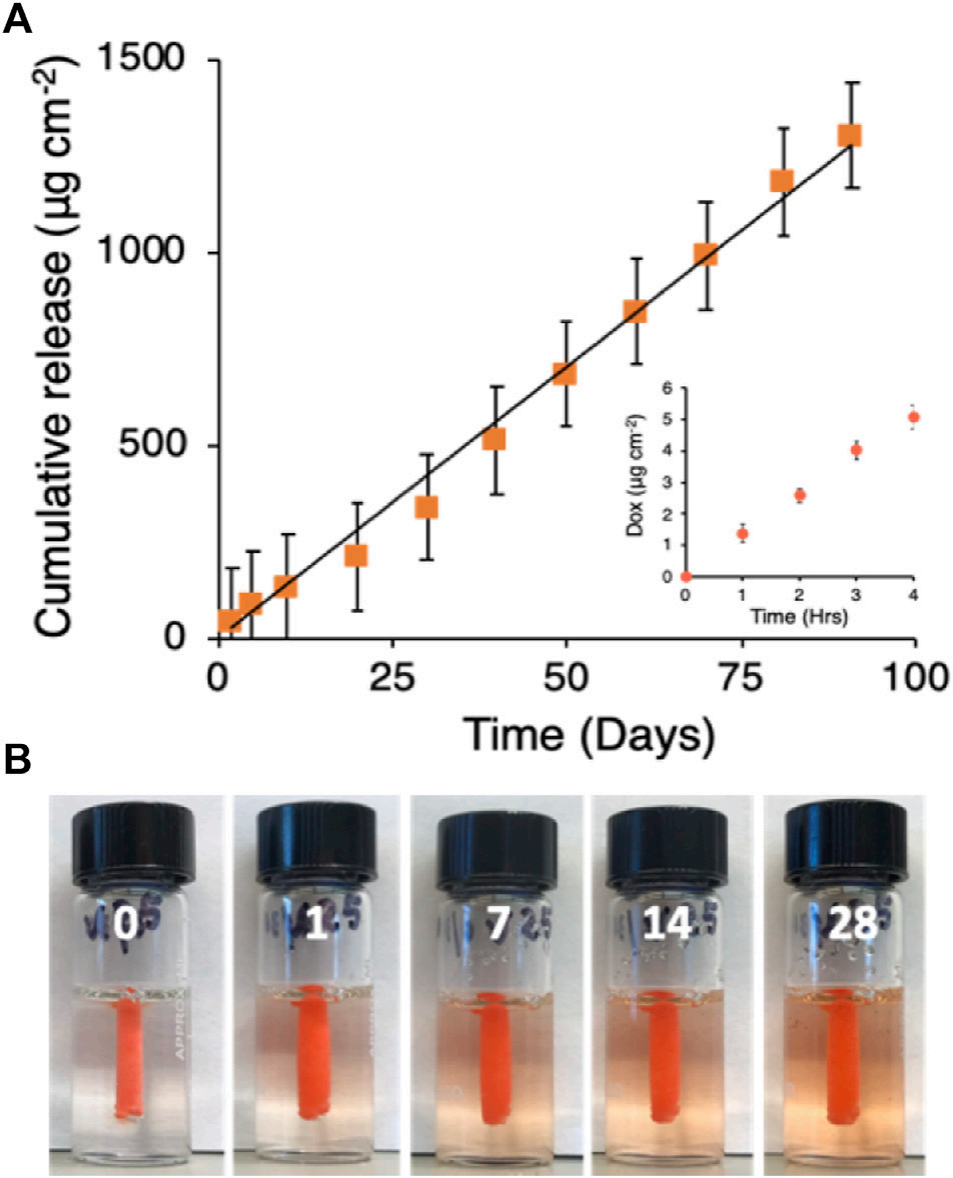
**(A)** Doxorubicin release kinetics. Black line is the linear fit to the mean data points (n = 3, *R* ^2^ = .99). Inset: first 4 h of release. **(B)** Visualization of DOX release from solid GPX into a gelatin tissue phantom over 28 days.

GPX350-DOX50 was prepared by dissolving the dry polyelectrolyte components in an aqueous solution of 50 mg mL^−1^ DOX HCl, a concentration near the maximum solubility of DOX HCl in pure water. DOX appeared to be homogeneously dissolved in the final GPX solution. Liquid GPX350-DOX50 (50 μL) was pipetted into a mini-dialysis cartridges (2 kD MW cutoff) and solidified by placing the cartridges in a 20-fold excess (1 mL) volume of normal saline. The saline was replaced hourly for the first 4 h and daily afterwards to maintain sink conditions. The DOX concentration was measured in the saline samples by UV-Vis spectrophotometry (485 nm). The cumulative release of DOX was linear (*R* ^2^ = .99) over 90 days at a rate of 14.8 ± .4 μg day^−1^ cm^−2^ of GPX350-DOX50 surface area (Figure 7A). There was no initial burst of release (Figure 7A inset). The cumulative release at 90 days was ∼14% of the total DOX loaded into GPX350-DOX50. The release of DOX into a gelatin tissue phantom is visible in Figure 7B.

The striking contrast between the release profiles of DOX compared to the non-ionic and non-aromatic iodinated contrast agents again suggested that DOX interacts strongly with the PG component of GPX. The iodinated contrast agents are entrapped in the micropores formed during GPX solidification and the release rate was limited by the rate of diffusion through the microporous matrix of solid GPX, resulting in presumably first-order release kinetics. The release rate of DOX, on the other hand, was limited in sink conditions by its low dissociation rate from PG (Cui et al., 2018) rather than diffusion through the microporous matrix, which resulted in zero-order release kinetics. A more detailed understanding of the rate-controlling release mechanism of aromatic compounds from GPX will require additional investigation. The results suggest that the release profile from GPX may be designed to be first or zero-order depending of the chemical characteristics of the drug.

Drug-eluting beads (DEBs) loaded with DOX have been approved since the early 2000 s (Lewis et al., 2016; Jia et al., 2022). Currently, there are four DEBs on the market: DC Bead™, HepaSphere™, Tandem™, and LifePearl™. All four have anionic functional groups, either sulfonates (DC Bead™ and LifePearl™) or carboxylates (HepaSphere™ and Tandem™), which are thought to interact with the single positive charge of DOX. The *in vitro* release profile of DOX has been compared for all four DEBs with qualitatively similar results (de Baere et al., 2016). Cumulative elution from DEBs loaded with 40 mg of DOX per ml of hydrated beads plateaued within 6 h, and at 24 h no further DOX had been released. The percent of the total DOX released was low; 20%–30% for the sulfonate DEBs and 6%–8% for the carboxylate DEBs. The results are consistent with other *in vitro* studies in which DOX release plateaued within hours with less than 30% of the DOX eluted and approximated diffusion-limited, first-order kinetics (Jordan et al., 2010; Ahnfelt et al., 2016). Comparison of GPX-DOX *in vitro* elution profiles to DEBs is problematic because elution profiles are dependent on numerous parameters (Gonzalez et al., 2008), including the shape and dimensions of the drug-eluting materials. The flat disc shape of the solidified GPX350-DOX50 samples used in the study have much lower surface to volume ratios than an ensemble of microspheres. The surface to volume ratio is a determining factor in release profiles, as evident in the greater release rate of DOX from smaller versus larger diameter DEBs (Ahnfelt et al., 2016). Nevertheless, although comparisons of the *in vitro* rate and extent or DOX release between DEBs and GPX-DOX are not meaningful, the distinct differences in elution kinetics, zero-order versus first-order, could be a significant factor to improve the effectiveness of TACE.

In general, zero-order release kinetics is a desirable property of a drug delivery system (Laracuente et al., 2020). It creates the potential of maintaining the drug concentration within the therapeutic window for prolonged periods, avoiding potential toxicity and repeated administration. In the case of a chemotherapeutic eluting embolic agent, this would mean maintaining for a prolonged period an effective concentration of drug in the surrounding tumor tissue to prevent proliferation of tumor cells that survive the ischemic shock of embolization. This may not be the case, however. Comparing DOX-loaded beads to unloaded (bland) beads in a rat model of hepatocellular carcinoma, Cortes et al. (2022) found that 3 days after embolization the bland bead-treated tumors had a greater percentage of tumor cells that survived the ischemic shock than the DOX-loaded beads. The stressed surviving cells may enter a quiescent state more refractory to treatment with anticancer drugs that disrupt the cell-cycle. At 7 days post-embolization there was no significant difference in the number of surviving tumor cells between the two groups. The authors concluded that the combination of embolic ischemia and DOX may be most effective shortly after embolization; the prolonged presence of DOX may not contribute significantly to TACE effectiveness. Comparative *in vivo* experiments in a suitable cancer model are required to determine if prolonged DOX delivery from GPX at a steady rate leads to better TACE outcomes than burst delivery from DEBs.

## 3 Conclusion

To summarize, GPX is a stable aqueous solution of oppositely charged polyelectrolytes that solidifies *in situ* through a novel mechanism based on the electrostatic condensation of oppositely charged polyelectrolytes in response to ionic strength gradients at the delivery site—a clinically and commercially practical solidification mechanism. The liquid-to-solid transition is not dependent on temperature differentials, polymerization reactions, covalent crosslinking of separate reactive components, or precipitation out of organic solvents, such as DMSO. Therefore, GPX can be packaged in single-barrel syringes, ready with minimal preparation for delivery through any standard single-lumen microcatheter. We have demonstrated that the viscosity of the liquid form can be tuned for the wide range of liquid embolic applications by adjusting any of several parameters, including the polyelectrolyte M_w_ and/or concentration. The composition and solidification mechanism are compatible with both permanent (metallic particles) or transient (iodinated compounds) contrast agents. Transient contrast allows GPX to be visible during the entire embolic procedure, but dissipates within 24 h avoiding interference with subsequent imaging. Doxorubicin can be mixed into the liquid form and is released without a significant burst and at a linear rate for greater than 90 days, a unique elution profile compared to DOX-eluting embolic beads.

## 4 Materials and methods

### 4.1 Materials

1*H*-Pyrazole-1-carboxamidine hydrochloride (21,678) was purchased from Chem-Impex International, 4-methoxyphenol (M0123) and triethylamine (TEA) (T0424) from TCI Chemicals, and N-(3-aminopropyl)methacrylamide hydrochloride (APMA·HCl) (21,200) from Polysciences, Inc. Anhydrous ethyl ether (EE) (E138-20), N, N-Dimethylformamide (DMF) (D131-4), acetone (A18-20), methanol (A412-20), glacial acetic acid (A38C-212), HPLC grade acetonitrile (A998-4) and TFA (A116-10X1) were purchased from Fisher Chemical. 4,4′-azobis (4-cyanovaleric acid) (V501) (11590) and sodium hexametaphosphate (96%, 305553) were purchased from Sigma-Aldrich. Methacrylamide (MAA) (L15013) was from Alfa Aesar, deuterium oxide (DLM-4–100) from Cambridge Isotope Laboratories, Inc., LiBr (151561) and NaCl (102892) from MP Biomedicals. Doxorubicin HCl (B0084-064210) was purchased from BOC Sciences. Iohexol (USP) and iopamidol (USP) were obtained from Divi’s Laboratories Limited. Tantalum (Ta) powder (TA-102) was purchased from Atlantic Equipment Engineers.

### 4.2 Synthesis of poly(N-(3-guanidinopropyl) methacrylamide) hydrochloride (pG·HCl) copolymers

Briefly, a flask was charged with *N*-(3-aminopropyl) methacrylamide hydrochloride (APMA·HCl) and the inhibitor 4-methoxyphenol (1 wt%, relative to APMA). DMF was added to dissolve APMA·HCl at 1 M concentration. TEA (2.5 equivalents) was added to the flask and the mixture was stirred for 5 min under N_2_ before 1 equivalent of *1H*-pyrazole-1-carboxamidine hydrochloride was added. The reaction proceeded at 20°C under N_2_. After 16 h, TEA·HCl salts were separated from the reaction mixture by vacuum filtration. The N-(3-guanidinopropyl) methacryamide (GPMA) monomer was extracted with diethyl ether 4 times and recovered as a dense oil. Finally, the monomer was dried under vacuum. The product was confirmed by proton and carbon NMR. ^1^H NMR (400 MHz, D2O): *δ* (ppm) 1.68 (q, CH_2_-CH_2_- CH_2_), 1.77 (s, CH_3_), 3.08 (m, CH_2_-N), 3.18 (m, CH_2_-N), 5.30 (s, =CH_2_), 5.55 (s, =CH_2_). ^13^C NMR: (400 MHz, D2O) *δ* (ppm) 17.74 (CH_3_), 27.62 (CH_2_), 36.62 (CH_2_-N), 38.71 (CH_2_-N), 121.13 (C=CH_2_), 138.83 (CH_2_=C), 156.6 2(C), 171.55 (C=O). GPMA was also verified by ESI mass spectroscopy (185.1 Da).

Random copolymers of GPMA·HCl and methacrylamide (MA) were synthesized by free radical polymerization. GPMA·HCl and MA monomers, at a 60:40 M feed ratio, were dissolved in a water methanol mixture at a total monomer concentration of 1 M. The free radical initiator 4,4′-azobis (4-cyanovaleric acid) was added at 1–5 wt%, depending on the target MW of the copolymer. The resulting mixture was septum sealed and degassed by bubbling with N_2_ for 1 h. The reaction proceeded under N_2_ with the temperature varied from 70°C–82°C depending on the target MW of the copolymer. The resulting solution was cooled, exposed to air, the polymer precipitated in acetone, then dissolved in water. The solution pH was adjusted to less than pH 6 with HCl. The resulting p (GPMA-HCl-co-MA) copolymers (Figure 1A) were purified by tangential flow filtration with deionized water. The polymer MW was characterized by aqueous size exclusion chromatography (SEC) on an Aglient HPLC 1260 Infinity equipped with refractive index detector and a Wyatt miniDAWN TREOS light scattering detector. The copolymer was ran in 1 wt% acetic acid in .1 M LiBr (pH = 3.3) at 1 mL min^−1^ on an Eprogen CATSEC 300 column. For MW analysis using light scattering, the dn/dc value for p (GPMA-co-MA) was determined using stock solutions of PG ranging from .25–2 mg mL^−1^. The mol% GPMA was determined by relative integration of the CH_2_-N groups (*δ* = 2.8–3.2 ppm) on GPMA (4 total H) and the saturated hydrocarbon groups (*δ* = .4–2.2 ppm) in the polymer backbone (5 total H’s on both GPMA and MA) and polymer sidechain (2 H’s on GPMA).

Poly (GPMA·HCl-co-MA) was also synthesized using an alternative method in which the *N*-(3-aminopropyl) methacrylamide hydrochloride (APMA·HCl) and methacrylamide (MA) were first copolymerized to create p (APMA·HCl-co-MA) followed by converting the primary amine sidechains to guanidinium·HCl groups. p (APMA·HCl-co-MA) was dissolved in water at a concentration of ∼1 M to which *1H*-pyrazole-1-carboxamidine HCl (1.15 equivalents of the amine sidechains) was added. The pH of the reaction mixture was increased to ∼9 with sodium carbonate. The reaction proceeded for 14–28 h under N_2_ at 25°C. Conversion of the APMA·HCl side chains to GPMA·HCl was >99% as determined by ^1^H NMR. The copolymer solution was acidified to pH < 6 with HCl and purified by tangential flow filtration with deionized water before lyophilization to produce the dry chloride salt. Equivalent results were obtained with both PG synthesis methods.

### 4.3 Preparation of sodium hexametaphosphate

Commercial sodium hexametaphosphate (Na_n_MP) is a mixture cyclic and linear of inorganic phosphate oligomers containing 10–20 P atoms per chain (Figure 1A) (Wazer and Holst, 1950; Rashchi and Finch, 2000). In their fully ionized form, cyclic inorganic polyphosphates have the formula (P_n_O_3n_)^n−^, while the linear form comprises (P_n_O_3n+1_)^n+2−^. The pK_a_ of the phosphate groups is 4.5 or less (Rashchi and Finch, 2000). Na_n_MP was dissolved in DI water, the pH was adjusted to 7.2–7.4 with NaOH, and lyophilized. For preparing GPX, the charge density of Na_n_MP at pH 7.2 was estimated to be one negative charge per phosphate group.

### 4.4 Preparation of GPX solutions

Solutions of (poly)GPMA·HCl_n_-*co*-MA (PG) and sodium hexametaphosphate (Na_n_MP) were prepared by adding water to dry mixtures of PG·HCl_n_ and Na_n_MP salts. The salts were mixed at 1:1 polymeric charge ratio, which corresponds to a mass ratio of 2.65:1 PG-HCl_n_ to Na_n_MP. Alternatively, the polymers were dissolved before mixing solutions at the same polymeric charge ratio with equivalent results. GPX solutions were prepared with PG·HCl_n_ concentrations ranging from 300–550 mg mL^−1^ and with PG-HCl_n_ copolymers having average MWs ranging from 19 to 53 kDa. The GPX solution nomenclature refers to the PG·HCl_n_ concentration, e.g., GPX350 was prepared with 350 mg mL^−1^ PG·HCl_n_. The Na^+^ and Cl^−^ counterion concentrations in the GPX solutions were estimated from the concentrations (mol L^−1^) and charge densities (mol g^−1^) of the polymeric salts, Na_n_MP and PG-HCl_n_, respectively, which corresponds to 3.5 mM NaCl per mg mL^−1^ of PG-HCl_n_. Hence, as examples, GPX350 and GPX550 comprise 1.26 M and 1.98 M NaCl, respectively. The majority GPX solutions were clear homogeneous solutions stable against macroscopic phase separation indefinitely. Some GPX solutions comprising low MW PG at the lower end of the concentration range separated into two distinct liquid phases (complex coacervation). These GPX solutions could be stabilized against phase separation by adding additional NaCl to the solution.

### 4.5 GPX with contrast agents and doxorubicin

GPX350 containing permanent metallic contrast agent (GPX350-Ta30) was prepared by mixing 30 wt% Ta particles into either dry PG·HCl_n_ and Na_n_MP before adding DI H20, or dispersing the Ta particles into the pre-dissolved GPX. Micronized tantalum metal powder (325 mesh) was sieved at 625 mesh to obtain particle sizes less than 20 microns. GPX350 solutions containing transient contrast agents (GPX350-IOH300 and GPX350-IOP300) were prepared by mixing dry iohexol or dry iopamidol powders with dry mixtures of PG·HCl_n_ and Na_n_MP salts, then dissolving the mixed powders in DI H_2_0. Alternatively, the dry mixed salts of PG·HCl_n_ and Na_n_MP were dissolved in commercial contrast media solutions, e.g., Omnipaque™ (iohexol), diluted appropriately with DI H_2_0 to achieve the final target mgI ml^−1^ concentration. GPX350-DOX50 was prepared by first dissolving doxorubicin·HCl in DI H_2_0 at 50 mg mL^−1^, near the maximum solubility in pure water. The dry salts of PG·HCl_n_ and Na_n_MP were dissolved in the doxorubicin solution. GPX350-DOX50-Ta30 was prepared by dispersing 30 wt% Ta particles into GPX350-DOX50.

### 4.6 Material characterization of GPX formulations

#### 4.6.1 Liquid state rheology

GPX solutions were prepared with PG·HCl_n_ copolymers with M_w_ ranging from 19 to 50 kDa and concentrations ranging from 350–550 mg mL^−1^. All solutions were prepared with Na_n_MP at a 1:1 polymeric charge ratio. Viscosities of the solutions were measured at 25°C using a Brookfield Amrtek DV2T viscometer with a small sample cup adaptor and CPA-41Z spindle. Flow behavior of the GPX formulations was characterized on a rheometer (AR 2000ex, TA Instruments) with a temperature-controlled deck using a 20 mm, 4° cone geometry at 25°C. A solvent trap prevented the sample from drying out during the experiment. The shear rate was stepped from 1 to 100 s^−1^. The flow behavior index (*n*) was estimated using the power law equation, *μ* = Κγ^n−1^, which relates viscosity (*μ*) to shear rate (*γ*). A solution with *n* < 1 shear thins, *n* = 1 for Newtonian solutions, and *n* > 1 indicates shear thickening. The wall shear rates (*γ*_w_) within the microcatheter, assuming laminar flow, were calculated using the expression *γ*_w_ = 4Q/πr^3^, where Q is the volumetric flow rate and r is the internal radius of the microcatheter.

#### 4.6.2 Injection force measurements

To measure injection forces, the GPX formulations were loaded into 1 mL syringes and connected to a stainless steel microcatheter (.0235″ ID, 120 cm length). The syringe was mounted on a syringe pump (PHD Ultra, Harvard Apparatus) to precisely control the flow rate. The force profile was recorded with a compression load cell (iLoad Mini, Loadstar Sensors) attached to the syringe pump between the driver and the syringe plunger. The formulations were injected at flow rates of .25, .50, 1.0, and 2.0 mL min^−1^ until a steady-state force was achieved. Experimental forces were converted to pressure using the bore diameter of the syringe (.19″, 4.8 mm).

#### 4.6.3 Solid state rheology

The viscoelastic properties of the GPX formulations in the solid state were characterized by injecting 1 mL of the GPX solution into excess normal saline to solidify. After 24 h, the solid sample was gathered and shaped into a sphere, then incubated in normal saline at 37°C for 24 h. The samples were placed on the temperature-controlled deck of the rheometer and subjected to frequency sweeps from .1 to 10 Hz, at .1% strain, at 37°C using a flat 20 mm geometry. Adhesive sandpaper was affixed to flat plate geometries to prevent slippage during measurements.

### 4.7 Transient contrast and DOX controlled release

The time course of non-ionic contrast agents diffusing out of solidified GPX in gelatin tissue phantoms was evaluated using micro-CT. Gelatin powder (Porcine skin Type A, 300 g bloom, 5 wt/v%) was heated in water to 45°C. Cylindrical tissue phantoms were created by adding the warm gelatin solution to a mold comprising a 2.5 cm diameter outside tube and a central interior 2 mm diameter tube. The tubes were lightly coated with olive oil to facilitate removal of the phantom. One end was sealed with paraffin and the warm gelatin solution was added to the outside tube. After cooling to room temperature, the central tube was removed leaving an empty 2 mm central tunnel in the solid gelatin cylinder. GPX400-IOHEX was prepared by dissolving dry PG-HCl_n_ (40 kDa, 400 mg/mL) and 1:1 Na_n_MP in iohexol or iodixanol solutions diluted with water to concentrations ranging from 0 to 270 mgI/mL. The GPX solutions (50 μL) were injected into the 2 mm diameter tunnel of molded gelatin cylinders. After GPX solidification, the gelatin phantoms were removed from the mold and wrapped in polyethylene film. The phantoms were imaged by micro-CT within 1 h of preparation and reimaged after 24 h.

GPX-DOX50 (50 μL) aliquots were pipetted into Float-A-Lyzer mini dialysis devices with MWCO = 2 kDa (Thermo Fisher Scientific), which were then capped and inserted into secondary vials containing 1 mL of 150 mM saline. GPX-DOX50 solidified within the dialysis compartment when contacted with saline. The samples were continuously mixed on a rotary shaker in an incubator at 37°C. At select time points, the 1 mL saline reservoir was replaced with 1 mL of fresh saline. Doxorubicin that had eluted into the reservoir was quantified by UV-vis spectrometry (485 nm).

## 5 Animal studies

### 5.1 Kidney selective embolization

All animal studies were approved by Institutional Animal Care and Use Committees. Swine are a commonly used model to test novel embolic agents. Arterial sites were accessed through the femoral artery using the Seldinger technique. From there, microcatheters were guided using standard techniques to renal arteries for selective embolization with GPX. Angiograms were captured before and after embolization using either the same microcatheter or a guide catheter. Angiography was performed immediately after embolization to assess the extent of the occlusion. In some cases, angiography was performed one and 7 days post-embolization to assess stability of the occlusion.

### 5.2 GPX in combination with coils

A Medtronic Axium™ Platinum Coil (8 mm × 20 cm) was placed in the renal artery, followed by angiography. GPX was then deployed proximal to the coil through a 4F Terumo Glidecath™ microcatheter, followed by angiography to access occlusion.

### 5.3 Porcine rete mirabile embolization

GPX-Ta30 was delivered to the Rete Mirabile through a Medtronic Echelon™ (2.1 F, .017″ ID) microcatheter, followed by angiography to assess occlusion.

### 5.4 Transient contrast with iohexol

GPX300-IOH300 was prepared by dissolving dry PG-HCl_n_ (300 mg mL^−1^) and Na_n_MP at a 1:1 polymeric charge ratio in a 300 mgI mL^−1^ solution of iohexol. Access to a sub-branch of the renal artery of a swine was obtained using a 4F catheter. GPX300-IOH300 (.3 mL) was delivered under fluoroscopy. After delivery, complete occlusion of the renal artery was confirmed by angiography. After 24 h, follow-up fluorography showed that radiopacity due to iohexol had completely dissipated out of the embolization site.

## Data Availability

The original contributions presented in the study are included in the article/Supplementary Material, further inquiries can be directed to the corresponding author.
